# Supplementary material for: Probiotic bacteria vs. yeast for gastrointestinal diseases treatment: Protocol for a systematic review and meta-analysis
Source: PLoS One. 2025 Jun 24;20(6):e0324926. doi: 10.1371/journal.pone.0324926 (PMC12186968; doi:10.1371/journal.pone.0324926)
Supplement: Attachment — Submitted filename: Rebuttal_Letter_PLOS_ONE.docx [file pone.0324926.s002.docx]

18 November 2024

Dear PLOS ONE Editorial Team,

**Re: Rebuttal Letter for Resubmission of Manuscript PONE-D-24-50095 (Previously PONE-D-24-39185)**

Title: Probiotic Bacteria vs. Yeast for Gastrointestinal Diseases Treatment: Protocol for a Systematic Review and Meta-Analysis

We appreciate the editor and reviewers for their valuable feedback on our manuscript. We have thoughtfully addressed each comment to enhance the clarity and thoroughness of our submission. Below, we present a point-by-point response to the reviewers’ comments and detail the revisions made. Page numbers in the response below refer to clean copy of the manuscript.

|  | **Comment** | **Response** | **Page** |
| --- | --- | --- | --- |
| **Comment 1** | "Following parameters should be included: Specific Gastrointestinal Diseases, Intervention Details, Outcome or Measures." | Thank you for highlighting the need for more detailed PICO elements. We have made the following revisions:  - Specific Gastrointestinal Diseases: We have revised the manuscript to explicitly list the gastrointestinal diseases included in this study, specifically focusing on (Irritable Bowel Syndrome (IBS), Inflammatory Bowel Disease (IBD), *Helicobacter pylori* infection, Crohn’s disease, Traveller’s diarrhoea, Antibiotic-associated diarrhoea and ulcerative colitis). This information is now clearly stated in the methods section.  - Intervention Details: The section on interventions has been expanded to include detailed descriptions of the probiotic types (bacterial vs. yeast), including strains, dosages, duration, and method of administration. These details can now be found in the Methods section under "Intervention" and "Comparator".  - Outcome or Measures: We have added a section detailing the primary and secondary outcome measures used to evaluate the intervention's effectiveness. This includes clinical symptoms, specific inflammatory markers, and quality of life assessments, as described in the Methods section "Outcomes". | 6-10 |
| **Additional Changes** | In addition to addressing the reviewer’s specific comments, we have also conducted a general review of the manuscript to improve clarity and flow where needed. | |  |

We appreciate the opportunity to resubmit our manuscript. We believe the changes made have strengthened it, and we hope it now meets the publication standards of PlosONE.

Sincerely,

Prof. Dr. Mohammed Abdullah Alshawsh
Adjunct Professor, Department of Pharmacology, Universiti Malaya, Malaysia
Senior Research Fellow, Monash University, Australia
Honorary Fellow, Murdoch Children's Research Institute, Australia
