# Supplementary material for: Probiotic bacteria vs. yeast for gastrointestinal diseases treatment: Protocol for a systematic review and meta-analysis
Source: PLoS One. 2025 Jun 24;20(6):e0324926. doi: 10.1371/journal.pone.0324926 (PMC12186968; doi:10.1371/journal.pone.0324926)
Supplement: Attachment — Submitted filename: Plos one response letter.docx [file pone.0324926.s003.docx]

28 March 2025

Dear Editor,

**Manuscript Title**: Probiotic Bacteria vs. Yeast for Gastrointestinal Diseases Treatment: Protocol for a Systematic Review and Meta-Analysis

**Manuscript ID**: PONE-D-24-50095

We sincerely thank the editor and reviewers for their constructive feedback. We have carefully revised the manuscript based on your comments. All textual changes are shown using Track Changes, and any updated or corrected references have been highlighted in yellow for easy identification. The line and page numbers in the response below refer to the track changes version of the revised manuscript.

Please find below our point-by-point responses to each comment.

Yours sincerely,

Prof. Mohammed Abdullah Alshawsh
Department of Pharmacology, Faculty of Medicine, Universiti Malaya

E: [alshaweshmam@um.edu.my](mailto:alshaweshmam@um.edu.my)

Monash University, Clayton VIC 3168, Australia
E: [mohammed.alshawsh@monash.edu](mailto:mohammed.alshawsh@monash.edu)

| **No** | **Reviewers Comments** | **Responses** | | | | | **Location in Revised Manuscript** |
| --- | --- | --- | --- | --- | --- | --- | --- |
| **Reviewer #1:** | | | | | | | |
| **-** | Dear Authors,  When I reviewed your article titled "Probiotic Bacteria vs. Yeast for Gastrointestinal Diseases Treatment: Protocol for a Systematic Review and Meta-Analysis" that you applied as a study protocol, I believe that it is a protocol that can be beneficial to the field of study, except for some minor corrections mostly related to the introduction part of your article. | Thank you for your positive feedback. We appreciate your comments and have addressed the minor corrections in the introduction as suggested. | | | | | **-** |
| 1 | Citation is required for the information you provided in lines 38-41 | Citations have been added (citation no. (3) and(4)) | | | | | Page 2  Line 42-44 |
| 2 | Your reference to the information you provided in lines 42-44 (citation number 3) is irrelevant. It should be cited with an appropriate study | The citation has been updated (citation no. (5)) | | | | | Page 2  Line 47 |
| 3 | Your reference to the information you provided in lines 44-47 (citation number 4) is a quote from the introduction part of the reference article. The referenced article wrote this information by referencing other studies. It would be more accurate if you also referenced these studies. Likewise, when citing a reference, it would be more appropriate in terms of scientific work ethics to cite the method or results of the referred study. | The citation has been updated (citation no. (6)) | | | | | Page 2  Line 50 |
| 4 | Your reference to the information you provided in lines 58-60 (citation number 6) is a quote from the introduction of the reference article. My previous comment is also valid for this criticism. | | Amended accordingly (citation no. (13)) | | | | Page 3  Line 64 |
| 5 | Your reference to the information you provided in lines 60-62 (citation number 7) is irrelevant. It should be cited with an appropriate study | | | The citation has been updated (citation no. (14)) | | | Page 3  Line 66 |
| 6 | Only one article was cited for the long and detailed information in lines 63-77. When the relevant reference was examined, it was seen that it did not fully cover the detailed information provided. The information provided should be supported with more appropriate articles by including the information in line 78 and cited | | | More relevant citations have been included (citation no. 15,16,17,18 and 19) | | | Page 3-4  Line 68-79 |
| 7 | The information provided in lines 93-34 is related to the fact that bacterial probiotics are more remarkable than probiotic yeasts as a supplement in gastrointestinal diseases. The relevant claim is a controversial issue, and citation no. 12 does not refer to this information. It should be cited with an appropriate article or the claim should be removed from the publication. | | | | The statement has been revised and a new citation has been added. | | Page 4  Line 98-100 |
| 8 | The sentence in lines 104-106 begins with "The purpose of this systematic review is...." Since this article is a study protocol article, the sentence should also start with the purpose of this study protocol. In addition, the rest of the sentence should be revised accordingly. | | | | | Amended accordingly | Page 5  Line 119-124 |
| **Reviewer #2:** | | | | | | | |
| 1 | Yes, the manuscript provides a valid rationale for the proposed study with clearly identified and justified research questions. The introduction outlines the academic problem gastrointestinal disorders and the role of probiotics in their management while emphasizing the need for a systematic review to compare bacterial and yeast probiotics. The research question is well-defined, focusing on the comparative effectiveness of these probiotic types, and is justified by the gaps in existing literature. The methodology is good, employing randomized controlled trials and critical appraisal tools to ensure reliability. | Thank you for your positive feedback. | | | | | - |
| 2 | Yes, But still it needs some clarification because the protocol does not explicitly mention whether a formal power analysis has been conducted to determine the required sample size for meaningful statistical comparisons. While meta-analyses often rely on available studies, an estimation of expected effect sizes and required study numbers would strengthen the methodological justification. Additionally, although heterogeneity is acknowledged using the I² test, further details on managing substantial heterogeneity (>65%), such as subgroup analyses or meta-regression, would be beneficial.  Lastly, the manuscript does not clearly indicate which analyses, if any, will be exploratory. Clarifying whether certain comparisons, such as probiotic strain-specific effects, are secondary or exploratory would enhance transparency. | Thank you for the comment. In meta-analysis of RCTs, a formal power analysis is not required in the same way it is for primary RCT studies. However, meta-analysis pools data from multiple RCTs, increasing the overall sample size and power. Instead of power analysis, sensitivity analyses (e.g., leave-one-out analyses) will be conducted to assess the robustness of findings by examining the impact of removing RCTs with small sample size. Since a meta-analysis with a small number of included studies may still yield meaningful results if effect size is large and confidence intervals are precise, small-study effects and publication bias will be considered if fewer than 10 RCTs are included in this systematic review. We have now clarified this in Section 2.8 (lines 305-310).  In Section 2.8 (lines 292-297), we described our strategy to manage high heterogeneity (>65%) by incorporating subgroup analyses based on strains, dose, duration, and disease type, and considering meta-regression if feasible.  Additionally, we explicitly stated that strain-specific comparisons (e.g., *Lactobacillus* vs. *Saccharomyces*) will be treated as exploratory analyses in (lines 304-305). These revisions have been made to improve clarity and methodological transparency. | | | | | Page 13-14 |
| 3 | Yes, however it is important addressing a few areas. First, it does not mention whether sample size estimation or power analysis has been performed, which is essential for ensuring the reproducibility and robustness of the study. Including a discussion on expected effect sizes or the number of studies needed would enhance the overall clarity and rigor.  Additionally, while the protocol suggests contacting authors to handle missing data, it would be useful to specify alternative methods, such as imputation strategies, in case responses are not obtained. | The sample size estimation and power analysis issues have been addressed in the revised manuscript (lines 305-310).  For missing data, while imputation methods like mean substitution and last observation carried forward (LOCF) are commonly used in primary studies, they are not generally recommended in meta-analysis, as imputing missing values can introduce systematic errors and lead to biased effect size estimates.  Instead, if author contact is unsuccessful after multiple attempts, alternative strategies will be employed, including conducting sensitivity analyses by excluding studies with missing data and comparing results with and without them. We will also use available summary statistics (e.g., estimating standard deviations (SD) from reported SEM, confidence intervals, interquartile ranges, or *p*-values if provided) to handle missing data. If missing data are more prevalent in certain subgroups (e.g., smaller studies or specific interventions), meta-regression will be used to assess whether missing data influence effect estimates. Finally, if key studies lack essential data and cannot be included quantitatively, their findings will be synthesized narratively rather than through quantitative analysis. This has been added page 16, lines 328-337  We also stated that attrition bias will be evaluated using the RoB-2 tool (line 270). | | | | | Page 14 & 15 |
| 4 | Yes, but the authors could specify where the final dataset will be deposited. This would strengthen compliance with open data requirements. | Upon completion of the systematic review and meta-analysis, the final dataset and analytical code will be deposited in an open-access repository such as Figshare, Dryad, or Zenodo to ensure accessibility and compliance with open data-sharing requirements (lines 367-372). | | | | | Page 17 |
| 5 | Yes, the manuscript is intelligible and written in Standard English, but minor grammatical and stylistic improvements would enhance clarity and readability. It would benefit from professional proofreading or language editing before submission. | We have now carefully edited the manuscript for grammar and typo mistakes. | | | | | Page  1-17 |
| **Reviewer #3:** | | | | | | | |
| 1 | The authors chose to apply “randomized clinical trials (RCTs)” in their study. They should at least define this approach in the background or upfront in the paper (not in the study design). They should also discuss about its pros and cons in brief. In this context, we already know that in RCTs, the two or more groups of people in each trial should be as similar as possible, except for the received treatments, to ensure that any difference in the outcomes between the groups are solely due to the treatments they received. Since the authors aim to consider participants of any age or any gender or any ethnicity in these trials, I suspect that they will end up with extremely small (n = 2 to 10) sample size in only one group (received treatment group) for a specific age or gender in a few cases. Do they think that applying RCTs will help in drawing a fair unbiased conclusion in those cases? In other words, how will they deal with selection bias in that case? Which statistics they propose to apply for that comparative analysis? | Thank you for your comment. We have added a brief statement for RCTs in the Introduction (lines 104–112), highlighting their pros (e.g., reduced bias through randomization) and limitations (e.g., limited generalizability and subgroup imbalances).  Regarding population diversity, we acknowledge potential subgroup variability (e.g., age, gender, ethnicity). To address this, subgroup analyses and meta-regression will be conducted where data permit, especially in cases of substantial heterogeneity (I² > 65%) as clarified in (lines 297-300). Additionally, sensitivity analyses will be conducted to assess the robustness of findings by examining the impact of removing RCTs (leave-one-out analyses) with small sample size (lines 305-310). These methods will help explore and mitigate selection bias in our pooled analysis. | | | | | Pages 4-5  &  14 |
| 2 | I understand that the authors do not intend to apply any statistics to deal with the missing data, since it may introduce biases itself. However, the authors’ original plan to contact the first authors of the published papers for this systematic review protocol, seems to be an incompetent approach to deal with the missing data, especially when the publications are older (for ex., 4 to 10 years old publications). This solution may work if they consider mostly recent publications. What will be the impact of publication bias in that case? Otherwise, how do the authors plan to avoid attrition bias? | As we mentioned in our response to the same comment from Reviewer #2, if author contact is unsuccessful after multiple attempts, alternative strategies will be employed, including conducting sensitivity analyses by excluding studies with missing data and comparing results with and without them. We will also use available summary statistics (e.g., estimating standard deviations (SD) from reported SEM, confidence intervals, interquartile ranges, or *p*-values if provided) to handle missing data. If missing data are more prevalent in certain subgroups (e.g., smaller studies or specific interventions), meta-regression will be used to assess whether missing data influence effect estimates. Finally, if key studies lack essential data and cannot be included quantitatively, their findings will be synthesized narratively rather than through quantitative analysis. This has been added page 16, lines 328-337  We also stated that attrition bias will be evaluated using the RoB-2 tool (line 270) and then sensitivity will be applied to avoid this type of bias. | | | | | Page 15-16 |
| 3 | “After the removal of duplicate articles, studies will be classified into three categories: relevant, irrelevant, or uncertain” – which criteria help to identify uncertain articles? Please clarify. | This has been addressed in the revised manuscript (Section 2.4, lines 239-242), stating that “uncertain” studies lack key details in the title/abstract (e.g., probiotic type, comparator, outcomes) and will undergo full-text review to determine eligibility. | | | | | Page 11 |
| 4 | there remains a gap in synthesizing the most effective strains and dosages, which this review aims to address” – I understand that the designed protocol may help to identify the best type of probiotics for different gastrointestinal disorders. How does this protocol help to select appropriate dosage of probiotics with the available sample sizes for a specific gender or age? | Thank you for the comment. While this protocol does not aim to determine exact dosing for specific subgroups, we will extract dosage-related data and, where feasible, conduct subgroup analyses or meta-regression based on age, gender, or disease type (Section 2.8, lines 292-297). This exploratory approach may reveal dosage-related trends but is not intended to define optimal doses for different demographics. | | | | | Page 13-14 |
| 5 | How does Review Manager help in the meta-analyses? Please clarify this section step by step in the manuscript for the general readers | We have added a new subsection titled “2.8.1 Meta-Analysis Procedure Using Review Manager” (lines 311-324) that outlines the steps of conducting meta-analysis using RevMan in a clear, step-by-step format. | | | | | Page 14-15 |
| **Minor concerns** | | | | | | | |
| 1 | Authors should briefly explain different tools or scores for the ease of general readers. For e.g., instead of mentioning only ‘Rayyan’ platform, the authors could write – “AI-powered ‘Rayyan’ platform, that is designed to streamline systematic literature reviews by identifying relevant studies quickly since it reduces screening time up to 90%”. | Amended accordingly | | | | | Page 11  Line 235-237 |
| 2 | In line no. 243 of page no.10, the authors mention about ‘number of arms’. What does it mean? | “Number of arms” refers to the number of intervention groups in a randomized controlled trial (e.g., two-arm trial compares two interventions) | | | | | - |
| 3 | How do the participant characteristics, i.e., the number of withdrawals with/without reasons (line 245 of page no. 10) help in this protocol? | Thank you for your observation. Reporting the number of withdrawals with or without reasons is important for assessing the risk of attrition bias and evaluating the overall quality and reliability of each study. This information helps ensure transparency and strengthens the interpretation of the pooled outcomes in the meta-analysis. | | | | | - |
| 4 | The background lacks adequate references against most of the statements from previous findings. In many cases, they did not even provide a single reference (e.g., line no. 52 to 54 of page 3). For instance, when the authors mention “a variety of studies” (line no. 39 to 41 of page 2) or “several clinical trials and experimental studies” (line no. 90 to 92 of page 4), they should at least provide two or three references against their statements. | Relevant citations have been added to line 44 ( citation no.4), line 47 - 79 ( citation no.5 to 19 ), and line 97 (citation no. 24, 25) | | | | | Page 1-4 |
