# Supplementary material for: Probiotic bacteria vs. yeast for gastrointestinal diseases treatment: Protocol for a systematic review and meta-analysis
Source: PLoS One. 2025 Jun 24;20(6):e0324926. doi: 10.1371/journal.pone.0324926 (PMC12186968; doi:10.1371/journal.pone.0324926)
Supplement: Attachment — Submitted filename: Plos_one_response_letter_auresp_2.docx [file pone.0324926.s004.docx]

23 April 2025

Dear Editor,

**Manuscript Title**: Probiotic Bacteria vs. Yeast for Gastrointestinal Diseases Treatment: Protocol for a Systematic Review and Meta-Analysis

**Manuscript ID**: PONE-D-24-50095R1

We sincerely thank the Academic Editor and all reviewers for their thoughtful comments and constructive feedback on our manuscript. Please find below our point-by-point responses to each comment.

Yours sincerely,

Prof. Mohammed Abdullah Alshawsh
Department of Pharmacology, Faculty of Medicine, Universiti Malaya

E: [alshaweshmam@um.edu.my](mailto:alshaweshmam@um.edu.my)

Monash University, Clayton VIC 3168, Australia
E: [mohammed.alshawsh@monash.edu](mailto:mohammed.alshawsh@monash.edu)

| **No** | **Reviewers Comments** | **Responses** | **Location in Revised Manuscript** |
| --- | --- | --- | --- |
| **Reviewer #1:** | | | |
| **1** | When I reviewed your revised article titled "Probiotic Bacteria vs. Yeast for Gastrointestinal Diseases Treatment: Protocol for a Systematic Review and Meta-Analysis" that you applied as a study protocol, again, I believe that it is a protocol that can be beneficial to the field of study. Except for my 3rd criticism (maybe you missed), I see the minor corrections that I suggested, mostly done. Therefore, I repeat my words: “The referenced article wrote this information by referencing other studies. It would be more accurate if you also referenced these studies. Likewise, when citing a reference, it would be more appropriate in terms of scientific work ethics to cite the method or results of the referred study.” I suggest one last minor correction that I suggested to you before. After that last correction, there won't be any additional criticism from me.  To remind my 3rd criticism (for the 1st version of your manuscript):"3- Your reference to the information you provided in lines 44-47 (citation number 4) is a quote from the introduction part of the reference article. The referenced article wrote this information by referencing other studies. It would be more accurate if you also referenced these studies. Likewise, when citing a reference, it would be more appropriate in terms of scientific work ethics to cite the method or results of the referred study." | Thank you. We have replaced the citation and added the original studies referenced in that article. These sources are now correctly cited in the revised manuscript. | Page 2  Line 50 |
| Journal Requirements: | | | |
| **1** | Please review your reference list to ensure that it is complete and correct. If you have cited papers that have been retracted, please include the rationale for doing so in the manuscript text, or remove these references and replace them with relevant current references. Any changes to the reference list should be mentioned in the rebuttal letter that accompanies your revised manuscript. If you need to cite a retracted article, indicate the article’s retracted status in the References list and also include a citation and full reference for the retraction notice. | Thank you for the comment. We have carefully reviewed all references and confirm that none have been retracted. In response to Reviewer #1’s suggestion, we have replaced citation No. [6] with citations [6–10]. |  |
